# Particle-Associated Microbial Community in a Subtropical Lake During Thermal Mixing and Phytoplankton Succession

**DOI:** 10.3389/fmicb.2019.02142

**Published:** 2019-09-13

**Authors:** Orna Schweitzer-Natan, Maya Ofek-Lalzar, Daniel Sher, Assaf Sukenik

**Affiliations:** ^1^Kinneret Limnological Laboratory, Israel Oceanographic and Limnological Research, Haifa, Israel; ^2^Department of Marine Biology, Leon H. Charney School of Marine Sciences, University of Haifa, Haifa, Israel; ^3^Bioinformatics Service Unit, University of Haifa, Haifa, Israel

**Keywords:** phytoplankton, heterotrophic bacteria, microbial community, particle-associated, lake microbiome

## Abstract

Ecosystem dynamics in monomictic lakes are characterized by seasonal thermal mixing and stratification. These physical processes bring about seasonal variations in nutrients and organic matter fluxes, affecting the biogeochemical processes that occur in the water column. Physical and chemical dynamics are generally reflected in seasonal structural changes in the phytoplankton and bacterio-plankton community. In this study, we analyzed, using 16S rRNA amplicon sequencing, the structure of the bacterial community associated with large particles (>20 μm) in Lake Kinneret (Sea of Galilee, Israel), and its associations to phytoplankton populations. The study was carried out during late winter and early spring, a highly dynamic period in terms of thermal mixing, nutrient availability, and shifts in phytoplankton composition. Structural changes in the bacterioplankton population corresponded with limnological variations in the lake. In terms of the entire heterotrophic community, the structural patterns of particle-associated bacteria were mainly correlated with abiotic factors such as pH, ammonia, water temperature and nitrate. However, analysis of microbial taxon-specific correlations with phytoplankton species revealed a strong potential link between specific bacterial populations and the presence of different phytoplankton species, such as the cyanobacterium *Microcystis*, as well as the dinoflagellates *Peridinium* and *Peridiniopsis*. We found that *Brevundimonas*, a common freshwater genus, and *Bdellovibrio*, a well-known Gram-negative bacteria predator, were positively associated to *Microcystis*, suggesting a potentially important role of these three taxa in the microbial ecology of the lake. Our results show that the dynamics of environmental abiotic conditions, rather than specific phytoplankton assemblages, are the main factors positively correlated with changes in the community structure as a whole. Nevertheless, some specific bacteria may interact and be linked with specific phytoplankton, which may potentially control the dynamic patterns of the microbial community.

## Introduction

Freshwater lakes are dynamic and complex ecosystems driven by variations in physical and chemical conditions across time and space. The dynamics of monomictic lakes are characterized by seasonal thermal mixing and stratification, nutrient and organic matter fluxes, light availability and biogeochemical processes that occur in the water column (Wetzel, 2001). Variations in thermal conditions and nutrient availability in the water body, as well as light intensity, are generally reflected in the structure of the phytoplankton community and affect seasonal phytoplankton succession (Lv et al., 2014; Rigosi et al., 2014; Filstrup et al., 2016). Seasonal phytoplankton succession in lakes, as described in the plankton ecology group (PEG) model (Sommer et al., 2012), is characterized during winter by a minimum biomass of species adapted to low light and temperature, a spring bloom of diatoms or green algae, and summer populations that vary in relation to the trophic status of the lake, including nitrogen-fixing cyanobacteria or diatoms.

Heterotrophic microbial communities, together with phytoplankton, play a prominent role in the structure and function of freshwater ecosystems, and may also dramatically change over time in relation to biotic and abiotic conditions (Niu et al., 2011; Song et al., 2017). Seasonal changes in bacterial community composition and activity are correlated with, and potentially driven by, physical parameters, such as temperature and pH (Yannarell and Triplett, 2005; Rösel et al., 2012). For example, water column thermal mixing (turnover) followed by a return to stratified conditions is a natural ecosystem disturbance that is known to influence the assembly of microbial communities (Shade et al., 2012; Garcia et al., 2013). Nutrient enrichment (eutrophication) is also considered to be a main driver of microbial community composition (Song et al., 2017). Heterotrophic bacterial communities may also be affected by the type and abundance of phytoplankton, including cyanobacteria (Woodhouse et al., 2016) and other phytoplankton (Niu et al., 2011). In a study of six north temperate humic lakes, over half of the variation in bacterial community composition could be explained by temporal patterns in the phytoplankton community, and covariation between changes in phytoplankton and the environment (Kent et al., 2007).

Several mechanisms may underlie the effect of phytoplankton on heterotrophic bacteria. First, the influence of phytoplankton on bacterial community may be through the release of dissolved organic matter and detritus, following algal cell death (Baines and Pace, 1991). Phytoplankton can negatively affect populations within the bacterial community through nutrient competition, antibiotic release and bacteriovory by mixotrophs (Nygaard and Tobiesen, 1993). However, Phytoplankton also provide refuge from predation for associated bacteria (Main et al., 2015), as well as a habitat for endophytic bacteria living within algal cells and epiphytic bacteria that live in the phycosphere surrounding algal cells (Cole, 1982). It is worth mentioning that many studies have focused on bacterial populations associated with cyanobacterial blooms, in order to better understand the process and mechanism of bloom formation and development (Berg et al., 2009; Bagatini et al., 2014; Woodhouse et al., 2016), as their harmful impact on the sustainability of aquatic ecosystems and water quality has been recently highlighted.

The bacterial community in the water column is divided into two distinct groups which clearly differ in their assemblages: free-living and particle-associated bacteria (Allgaier and Grossart, 2006). Additionally, bacterial assemblages on particles may be strongly influenced by the composition and heterogeneity of the particles. Inorganic particles, phytoplankton and zooplankton all host different bacterial communities, and differences in bacterial community assemblages may also be observed between different zooplankton or phytoplankton organisms (Grossart et al., 2009; Woodhouse et al., 2016; Bižic-Ionescu et al., 2018a; Zäncker et al., 2019). Tang et al. (2009) suggested, that the trophic state of the water and the physicochemical properties of organic aggregates play a key role in sustaining the structure of attached bacteria. Particle-associated bacteria may play an important role in the remineralization of both dissolved and particulate organic matter (Tang et al., 2009; Parveen et al., 2013). These bacteria are generally considered ‘particle specialists’ due to their ability to degrade complex bio-macromolecules (Cottrell and Kirchman, 2000) and to consume algal-derived metabolites (Pinhassi et al., 2004). In the context of their interaction with phytoplankton, physical attachment of bacteria to the surface of microalgae may therefore suggest a tight functional association, positively or negatively affecting algal growth.

Lake Kinneret (Sea of Galilee, Israel) is subject to a Mediterranean climate, with hot dry summers and cool wet winters, with rainfall and main water and nutrient inflows limited to a period of 4–6 months of the year (October to April). The lake is subtropical, warm, monomictic meso-eutrophic, and is stratified annually from March-April until December-January. Shifts in the annual composition patterns of phytoplankton species are common in the lake since the mid-1990s, with modified patterns of blooming species in the spring and the appearance and establishment of toxin-producing cyanobacteria during winter and summer. While these unique phytoplankton patterns have been well studied and documented (Berman et al., 1992; Zohary, 2004; Zohary et al., 2014), the structure and dynamics of the heterotrophic bacterial populations associated with the phytoplankton in the lake are mostly unknown.

In this study we explored the structure and dynamics of the particle-associated microbial community in Lake Kinneret, focusing on large particles (>20 μm), collected across the photic layer of the lake. We analyzed temporal variations in the heterotrophic bacterial community, and aimed to identify potential taxon-specific interactions of heterotrophic bacteria with specific-phytoplankton species. We hypothesized that the particle associated bacterial populations are affected by the phytoplankton species and biomass, asking the following questions: (1) Does the bacterial community vary over time? (2) How are these variations related to environmental conditions and phytoplankton populations? (3) Are certain bacterial populations potentially associated with specific phytoplankton species? We focused on the winter spring period, when the lake was thermally mixed and rich in nutrients, and the structure of the phytoplankton community was expected to rapidly respond to these changes.

## Materials and Methods

### Sampling

Water samples were collected weekly in Lake Kinneret (32°50N, 35°35E) during the winter–spring period of 2015, starting during early January and ending in late April. Depth integrated water samples were collected from the epilimnetic photic layer (0–15 m) at the central monitoring station (Station A, the central deepest point of the lake). A 20 μm plankton net was used to collect and concentrate suspended particles (phytoplankton, zooplankton and non-living suspended particles) into 1L bottles. Samples were stored in a dark cooling container until further processing in the laboratory, 3–5 h after collection. The plankton net was washed with distilled water after each sample collection, to avoid cross-sample contamination. A volume of 50 ml from each sample was further filtered through a 5 μm polycarbonate membrane to collect the DNA of particle-associated bacteria. Filters were suspended in 1 mL preservation buffer (EDTA 40mM, Tris 50 mM pH = 8.3, Sucrose 0.75 M), to avoid DNA degradation, and stored at −80°C until further use. Data on chemical and physical parameters were collected by the Kinneret Limnological Laboratory (IOLR) and included measurements of water temperature, turbidity, dissolved oxygen, pH, nitrite, nitrate, ammonia and total dissolved phosphate (TDP), measured at several standard depths, and averaged over the upper 15-m layer. The chemical variables were measured following the standard methods described by Nishri (2011). Measurements of water temperature were obtained by the use of a Minos X CTD profiler (AML Oceanographic).

### Quantitative Analysis of Phytoplankton

Subsamples were fixed with Lugol’s iodine for microscopic observation and phytoplankton counting. Phytoplankton quantification was determined by the Utermohl’s sedimentation method (Utermohl, 1958) using an inverted optical microscope (Axio Observer, Zeiss, Germany) and further image processing by the ImageJ package (Collins, 2007). The biomass of phytoplankton in the >20 μm size range was estimated by calculating the projected area of sedimented phytoplankton objects per cubic meter of water (mm^2^/m^3^). The projected area calculation method was found to correlate well with biovolume in estimation of phytoplankton populations, and enables species-specific as well as a more global analysis (Rodenacker et al., 2006), thus it presents a suitable tool for phytoplankton investigation and was used here to quantify its population.

### DNA Extraction and PCR Amplification

DNA extraction was performed robotically at the Genomics Center, Biomedical Core Facility at the Technion (Israel Institute of Technology). The first step of extraction included the following chemical and mechanical treatments: samples were thawed, centrifuged for 10 min at 15,000 × *g* and the buffer was removed. Next, a lysis buffer (20 mM Tris⋅Cl, pH 8.0, 2 mM sodium EDTA, 1.2% Triton^®^ X-100) (DNeasy Blood & Tissue Kit, Qiagen) was added and the samples were mechanically pounded with two 3 mm stainless steel beads at a speed of 30 Hz for 1.5 min using TissueLyser LT (Qiagen). Thirty μL of lysozyme were then added and the tubes were incubated for 30 min at 37°C. After this, 25 and 200 μL of proteinase K and buffer AL of Qiagen were added, respectively, and the tubes were incubated at 56°C for 1 h with agitation. Finally, the tubes were centrifuged for 10 min at 5,000 × *g* and the upper liquid was transferred to a new 2 mL Eppendorf tube for further extraction by the Qiacube robot, via the DNeasy Blood & Tissue Kit. DNA samples were quantified with the PicoGreen dsDNA quantitation assay (Invitrogen, Burlington, ON, Canada). The yielded DNA concentrations ranged between 7.2–52.3 ng/μL.

Quantification of *Microcystis* 16S rRNA gene in the samples was performed by quantitative PCR using a Rotor Gene 6000 Real-Time PCR machine (Corbett Research, United Kingdom) by the SYBR^®^ Green approach. Primers set of forward CH (5′-AGCCAAGTCTGCCGTCAAATCA-3′) and reverse CI (5′-ACCGCTACACTGGGAATTCCTG-3′) were employed, targeting the *Microcystis* 16S rRNA gene (Conradie and Barnard, 2012) for a 102 bp amplification product. Total DNA extracted from each sample (concentration of 0.5 ng/μL) was used as a template and a negative control was included with the same reaction system and MilliQ water (Purelab, Elga, Germany) as a template. Each sample was quantified in duplicates. Serial dilutions of purified 16S rRNA gene PCR products from *Microcystis aeruginosa* KLL-C004 strain were used to generate a standard curve for positive controls. The thermocycling steps for qPCR amplification and calibration standard curves for positive controls were: Denaturation for 4 min at 95°C, followed by 35 cycles of 94°C for 30 s, 58°C for 30 s, and 72°C for 30 s. After amplification, a 7-min elongation step at 72°C was included. The qPCR reactions were performed in 25 μL system, including: a 2.5 μL qPCR buffer (50 mM Tris-HCL pH 8.3, 500 μg/ml BSA, 0.5% Ficoll, 1% Sucrose, 30 mM KCL, 3 mM MgCl), 1 μL of 10 mM dNTPs solution, 1.66 μL SYBR Green, 16.3 μL H_2_O, 0.5 μL Taq polymerase (Pluthero, 1993), 2 μL template DNA and 0.5 μL each primer. The obtained 16S rRNA gene copy number was normalized to copies/mL.

DNA samples were prepared for high-throughput sequencing using a two-step standard PCR protocol, as described by Green et al. (2015). Total DNA (concentration of 0.5 ng/μL) was used as a template for the first PCR amplification of partial 16S rRNA gene using the following primers targeting the V3–V4 variable regions: CS1_341F (5′-ACACTGACGACATGGTTCTACANNNNCCTACGGGAGGC AGCAG-3′) and reverse CS2_806R (5′-TACGGTAGCAGAGAC TTGGTCTGGACTACHVGGGTWTCTAAT-3′). The primers contained 5′ common sequence tags (known as common sequence 1 and 2, CS1 and CS2, respectively) as described by Moonsamy et al. (2013). PCR amplifications were performed in triplicate for each DNA sample, in 25 μL reactions using 96-well plates. A master mix for the entire plate was made using MyTaq Red Mix (BioLine, London, United Kingdom). Two μL of diluted DNA (0.5 ng/μL) was added to each PCR reaction. PCR cycling conditions were: 95°C for 5 min, followed by 28 cycles of 95°C for 30 s, 50°C for 30 s and 72°C for 1 min. A 5-min elongation step was performed at 72°C. Reactions were verified to contain specific amplification by agarose gel-electrophoresis. Following the first PCR, triplicate samples were combined, and the products sent to the DNA Services Facility of the University of Illinois, Chicago, where a second PCR was performed to incorporate barcodes and sequencing adapters (CS1 and CS2). Briefly, a second PCR amplification was performed in 10 μL reactions in 96-well plates to incorporate Illumina sequencing adapters and sample-specific barcodes. A mastermix for the entire plate was made using 2X AccuPrime SuperMix II (Life Technologies, Gaithersburg, MD, United States). Each well received a separate primer pair from the Access Array Barcode Library for Illumina sequencers (Fluidigm, South San Francisco, CA, United States; Item# 100−4876). Cycling conditions were: 95°C for 5 min, followed by 8 cycles of 95°C for 30 s, 60°C for 30 s and 68°C for 30 s. A final, 7-min elongation step was performed at 68°C. Samples were pooled in equal volume and purified using solid phase reversible immobilization (SPRI) cleanup, implemented with AMPure XP beads (Beckman Coulter, Brea, CA, United States) at a ratio of 0.6X (v:v) SPRI solution to sample. Final quality control was performed using TapeStation2200 (Agilent Technologies, Santa Clara, CA, United States) and Qubit analysis (Life Technologies). The pool was loaded on a MiSeq v3 flow cell (Illumina, San Diego, CA, United States) at a concentration of 5.5 pM and sequenced in 2 × 300 bp paired end format using a 600 cycle MiSeq v3 reagent cartridge. Library preparation was performed at the DNA services (DNAS) facility, within the Research Resources Center (RRC) at the University of Illinois at Chicago (UIC); sequencing was performed at the W. M. Keck Center for Comparative and Functional Genomics at the University of Illinois at Urbana–Champaign. Raw sequences were deposited in the Sequence Read Archive (SRA) of the National Center for Biotechnology Information (NCBI) database under the BioProject ID: PRJNA543623.

### Sequence Analysis and OTU Picking

MOTHUR (version 1.37.4) (Schloss et al., 2009) was used for quality control of the data of all samples. Paired reads were assembled into contigs using the make.contigs command (with default parameters, except for deltaq = 4 and PHRED score = 30). Low quality contigs were removed (containing ambiguities, homopolymers longer than 8 or sequences of length below 445 bp or longer than 470 bp) using the command screen.seq. Suspected chimera were detected using the chimera.uchime command, with the silva.gold.align database as reference, and removed. The filtered sequence dataset was clustered at the level of 97% sequence similarity, using the pick_otus.py command with default parameters in Qiime (version 1.9.1) (Caporaso et al., 2010). Representative sequences for each OTU were obtained by QIIME pick_rep_set.py script with default parameters. Based on the sequence of those representatives, OTUs were taxonomically classified using MOTHUR classify.seqs command with silva.nr_v128.align as reference (Yilmaz et al., 2014). OTUs assigned as Eukaryota, Archaea, Chloroplast or Mitochondria were removed. In total 850,987 sequences were retained (mean per sample = 56,732 ± 9820), clustered into 4,764 OTUs. For further analysis, OTUs classified as Cyanobacteria were removed, to retain heterotrophic populations. Relative abundance of Cyanobacteria sequences among the samples ranged between 38–64%. Following this filtration step, the data set was normalized by subsampling to 11,500 contigs per sample, using the ‘*rrarify’* algorithm provided with the ‘vegan’ package (version 2.5.5) (Oksanen et al., 2011) in R (version 3.4.3).

### Statistical Analysis

Alpha-diversity indices of heterotrophic communities were calculated for each sample: number of observed OTUs, Shannon index of diversity, and Simpson Dominance index using R package ‘vegan.’ The subsampled OTU data set was used to generate an ordination plot by non-metric multidimensional scaling (NMDS) based on Bray-Curtis dissimilarity, for exploring the temporal patterns of the heterotrophic microbial community dynamics. NMDS analysis was performed using the *metaMDS* algorithm in ‘vegan’ package (subsampled data was square-root transformed before being subjected to a Wisconsin double standardization). The environmental parameters correlating with the community composition were fitted using the ‘*envfit’* algorithm provided with the ‘vegan’ package. The significance of the associations was determined by running 999 random permutations.

Data used for exploring the composition and structure of the heterotrophic microbial community included the 100 most abundant OTUs among all samples (which comprised 82% of Heterotrophic reads), in order to avoid noise associated with low numbers, and to reduce the number of further tested microbial and metadata interactions. We used the K-means partitioning algorithm as an independent way for examining the microbiome structure and its behavior, implemented with the function ‘kmeans’ from the ‘stats’ package in R, after log2 transformation of the normalized OTU table. The partitioning of the 100 most abundant OTUs into different number of groups (K, tested between 2 and 12 groups) was tested using the resampling approach (Ben-Hur et al., 2002), using the Bioconductor package ‘clusterStab’ (Smolkin and Ghosh, 2003), whereby randomly selected subsets of OTUs were repeatedly clustered and the extent of similarity between the resulting clusters was examined.

The Spearman correlation was used to test significant associations (*p* < 0.05) between limnological variables and the heterotrophic bacterial OTUs, at the cluster level and at the OTU level, based on the relative abundance using the ‘psych’ package (Revelle, 2013).

## Results

### General Features of the Limnological System

#### Thermal Structure and Physical-Chemical Parameters

**FIGURE 1:**
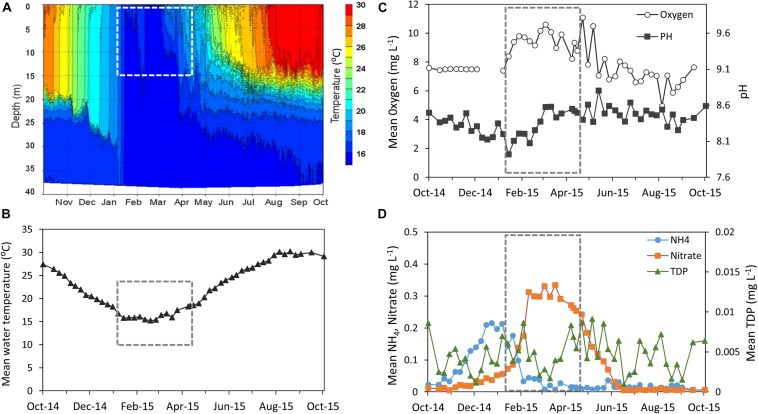
Abiotic conditions measured over the hydrologic year 2015 (October-14 to October-15) (Supplementary Table S1). **(A)** Changes in water temperature as a function of depth. **(B–D)** Physical and chemical measurements of the photic layer (depth average of 0–15 m): **(B)** water temperature (°C). **(C)** Oxygen (mg L^–1^) and pH **(D)** Ammonia, Nitrate, and total dissolved Phosphate (TDP) (mg L^–1^). The study period is framed by a dashed line. Data were obtained from the Lake Kinneret data center (IOLR), courtesy of Dr. W. Eckert, Dr. Y. Beeri-Shlavin, and M. Shlichter (Kinneret Limnologic Laboratory, IOLR).

Lake Kinneret showed dynamic changes in many limnological parameters over the hydrological year, including water temperature, dissolved oxygen, pH and nutrient abundance (Figure 1 and Supplementary Table S1). The chosen study period can be characterized as a highly active one, in which changes in phytoplankton assemblage are imposed by thermal mixing of the lake and enhanced nutrient loads due to winter floods followed by biogeochemical processes. The thermal structure of the water body (Figure 1A) shows that the lake was stratified until early January, when a full thermal mixing of the water body began which lasted until early March. Mean water temperature of the upper layer of the water (0–15 m, representing the photic layer) was minimal in late February (15°C) and gradually increased up to 19°C by the end of April (Figure 1B) as thermocline was established. Increasing pH values and relatively high dissolved oxygen concentrations were observed during the study period, reflecting the photosynthetic activity of a growing phytoplankton community (Figure 1C). Nutrient concentrations in the photic layer showed a typical seasonal pattern for Lake Kinneret, with a shift in the nutrient regime from ammonia to nitrate being the main N source (Figure 1D). Ammonium (NH_4_) concentration in the photic layer (0–15 m depth) rose to 0.21 mg/L on early January and then declined due to biological assimilation and oxidation to nitrate via nitrification. A temporary increase in nitrite concentration (Supplementary Figure S1) indicated the contribution of the nitrification process. Subsequently nitrate concentration increased due to both nitrification and winter loads from the lake’s watershed, and reached 0.33 mg (N) L^–1^ in mid-March. Dissolved phosphate concentrations peaked at 0.009 mg L^–1^ on early February, declined to 0.002 mg L^–1^ in mid-March, and rose again by the end of April.

#### Phytoplankton Succession

**FIGURE 2:**
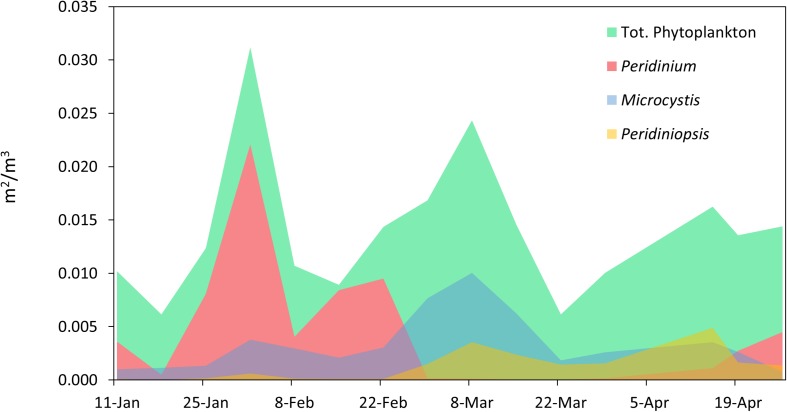
Temporal variations in phytoplankton biomass and community composition. The biomass of total phytoplankton and of populations of *Peridinium*, *Microcystis*, and *Peridiniopsis* was determined by calculating the projected area of the sedimented phytoplankton objects observed under the microscope per cubic meter of water, using image processing with the ImageJ package.

During the study period the phytoplankton community was comprised predominantly of three major taxa (the dinoflagellates *Peridinium* and *Peridiniopsis* and the cyanobacterium *Microcsytis*), which alternately dominated the photic zone of the water column (Figure 2). Three succession events in the phytoplankton community were observed during the study period. The first event occurred in late January as the biomass of *Peridinium* increased, comprising over 70% of the total biomass of phytoplankton by early February. The *Peridinium* population collapsed over several weeks and was below detection limit by the end of February. A second event started in late February and was dominated by a moderate biomass increase in *Microcystis* and *Peridiniopsis*, respectively comprising 40 and 15% of the total biomass. The two populations reached maximal biomass by early March. A biomass increase of *Peridiniopsis* was recorded again in mid-April, with a reduced presence of *Microcystis. Peridinium* population reappeared in Late April, though at a much lower biomass than the one recorded in February (Figure 2).

To verify the results of the microscopic counts, Real-Time quantitative PCR (qPCR) amplification of the *Microcystis* 16S rRNA gene was employed to quantify and compare the *Microcystis* population among samples. *Microcystis* is known to have two copies of rRNA gene in its genome (Kaneko et al., 2007) and in many cases ploidity was reported in cyanobacteria (Griese et al., 2011). However, this does not affect the results as gene abundance is reported in the context of seasonal trends in *Microcystis* population. Therefore, the absolute number of cells based on cellular gene quota was not calculated. Nevertheless, the 16S rDNA gene copy numbers, measured by qPCR, and the bio-volume estimation based on microscopic counts, have similar temporal trends (Supplementary Figure S2).

### Bacterial Community Composition

16S rRNA amplicon sequencing revealed that, over the study period, Cyanobacteria was the most abundant phylum among the particle-associated bacterial community, accounting for 38–64% of the total sequences found. *Microcystis* sp. dominated the cyanobacterial population, and was the primary component of the particle-associated community, comprising 44% of the total sequences. *Synechococcus* sp. and *Aphanizomenon* sp. were also found among the cyanobacterial population, respectively accounting for 3.2 and 2.6% of the total sequences (Supplementary Table S2). The 10 most abundant OTUs across all samples comprised over 70% of the sequences, including abundant lake bacteria belonging to α-Proteobacteria and γ-Proteobacteria. These results indicate that the bacterial community is dominated by a few abundant taxa, with a large number of relatively rare OTUs, many of which were found only in a limited number of samples (Supplementary Figure S3).

#### Heterotrophic Bacteria Composition Varies Over Time

**FIGURE 3:**
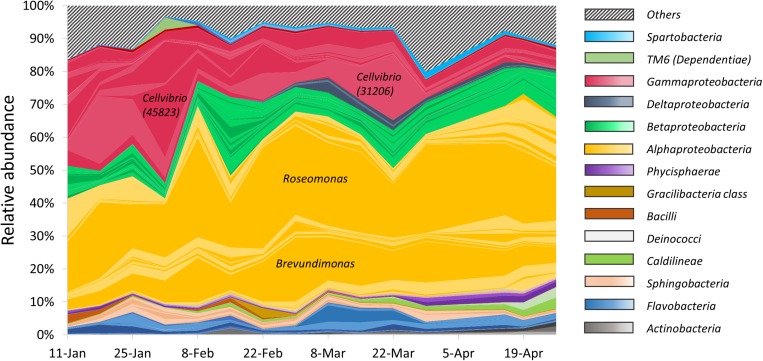
Relative abundance of particle-associated heterotrophic bacteria at class level obtained from 16S rRNA gene sequencing. Colored areas represent the 100 most abundant OTUs. Each colored line represents a single OTU. Striped area represents the rest of the heterotrophic OTUs.

Heterotrophic bacteria composition showed a dynamic pattern over the study period (Figure 3). The most abundant phylum was Proteobacteria, with a remarkable dominance for the class α-Proteobacteria. The structure of the α-Proteobacteria group was relatively stable, with the population dominated by two main OTUs throughout the study period, identified as *Roseomonas*, and *Brevundimonas*. These two OTUs accounted, respectively, for up to 30 and 20% of the total heterotrophic reads. In contrast, many members of the class γ-Proteobacteria showed a highly dynamic pattern and included two OTUs affiliated as *Cellvibrio* (OTU 45823 and OTU 31206). These two members accounted, respectively, for up to 18 and 21% of the total heterotrophic reads, and appeared to alternate in high relative abundance over time, as the first one peaked on early February and the second peaked on late March. The relative abundance of γ-Proteobacteria decreased throughout the study period, and constituted a minor component during late March and April, as thermal stratification was established. Alpha diversity indices of the samples (Supplementary Table S3) exhibited moderate changes over the entire study period. The bacterial richness (S) varied from 427 to 880 OTUs, Shannon’s index ranged between 3.4 and 4.8 and the Simpson index was between 0.89 and 0.98. A minor change in diversity was found in the sample collected on 15/02/15, 2 weeks after the *Peridinium* bloom event, when the three indices pointed to a more diverse community (S = 868, Shannon = 4.82 and Simpson = 0.98), suggesting that the collapse of the bloom may have caused an increase in bacterial diversity.

#### The Heterotrophic Microbial Community Composition Is Mainly Associated With Abiotic Factors

**FIGURE 4:**
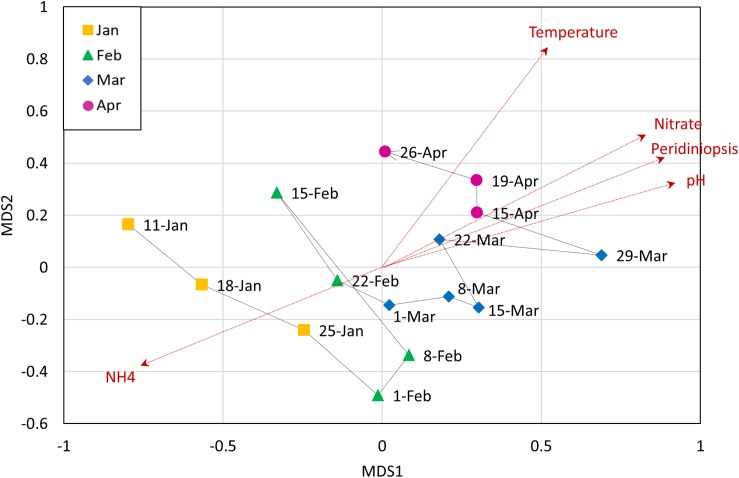
Non-metric multidimensional scaling (NMDS) analysis of heterotrophic community composition of data from OTU abundance, with corresponding environmental variables. Analysis was based on Bray-Curtis dissimilarities. Vectors are environmental variables that were found to be significantly affecting the distribution of the points (dashed red arrows). The line connecting the data points represents the temporal change in the community.

Non-metric multi-dimensional scaling (NMDS) analysis of the OTUs abundances was used to examine the contribution of potential explanatory environmental variables to the heterotrophic community composition, by fitting environmental vectors to the ordination (Figure 4). We found a significant correlation between the ordination pattern of the bacterial community and two opposite abiotic parameters: ammonia and pH (*r* = 0.6824 and *p* = 0.002; *r* = 0.6747 and *p* = 0.001, respectively). Temperature, nitrate, oxygen and *Peridiniopsis* were also significantly correlated with the bacterial community ordination (Supplementary Table S4). As shown in Figure 4, the direction of which ammonia corresponds to the heterotrophic community is inverse to that of other environmental vectors, suggesting that it may be a key player in shaping the heterotrophic community. No significant correlation was found with *Microcystis* and *Peridinium* biomasses (Supplementary Table S4), therefore we suggest that the changes occurring within the whole heterotrophic community are mostly driven by abiotic parameters, mainly by pH and ammonia.

### Microbial Community Structure and Dynamics

**FIGURE 5:**
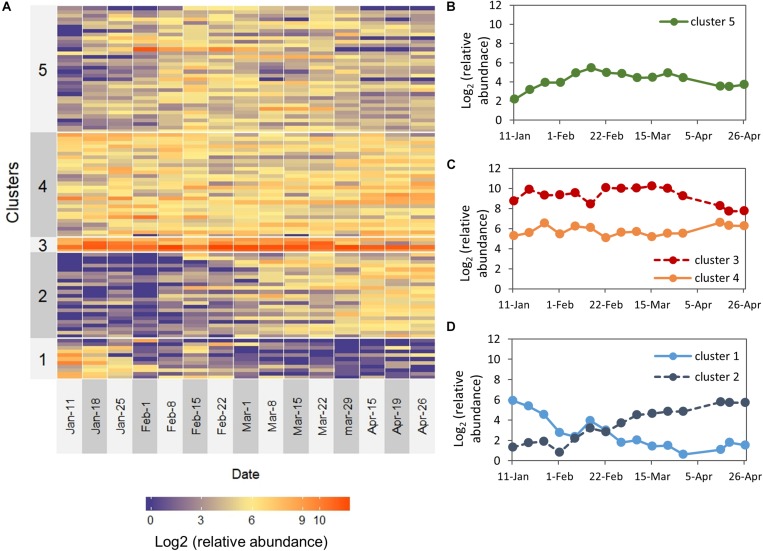
Temporal patterns for the 100 most abundant OTUs over the study period. **(A)** The heat map depicts the log2 (relative abundance) for each OTU. OTUs are ordered based on K-means clustered into five groups (left labels). **(B–D)** Plots of the temporal dynamics of clusters (centroids) that were visually assigned to: **(B)** OTUs found at intermediate abundance in the middle of the study period, **(C)** high abundance and intermediate abundance throughout the entire study period **(D)** descending abundance and ascending abundance.

We next aimed to identify groups of OTUs sharing similar temporal dynamics. K-means partitioning demonstrated five distinguishable groups that differed in their temporal patterns (Figure 5A): descending abundance (cluster 1), ascending abundance (cluster 2), high abundance throughout the study period (cluster 3), intermediate abundance throughout the study period, (cluster 4), and OTUs found at intermediate abundance in the middle of the study period (cluster 5). These clusters reflect differences in both temporal patterns and relative abundance (Figures 5B–D). Determination of *K* = 5 was based on subjective estimation, although it was not supported by high statistical significance.

Cluster 1 (descending abundance) was characterized by relatively high abundance during January and a low abundance between February–April, and contained several taxa which are known to be involved in methane metabolism, including *Methylomonas, Methyloparacoccus*, and *Methylotenera* (Beck et al., 2013). *Nitrosomonas*, known to take part in nitrogen cycling (Burrell et al., 2001), also belonged to this group.

Cluster 2 (ascending abundance) was characterized by low abundance between January-February and a higher abundance during March–April. α-Proteobacteria was the dominant class in this group and among its members were several OTUs belonging to the order *Rickettsiales*, known to be endosymbionts and even pathogens of eukaryotes, as well as a member of the LD12 subclade, the freshwater sister group to marine SAR11. *Peredibater*, a member of the BALOS group of predatory bacteria (Bdellovibrio and-like organisms) was also found in this group and exhibited a clear dynamic pattern with zero reads during January-February and high abundance during March-April.

Cluster 3 (the high abundance group) contained the α- and γ-Proteobacterial OTUs described earlier (*Bervundimonas*, *Roseobacter*, *Cellvibrio*) and a Xanthomonadacae. *Brevundimonas* and members of the Xanthomonadaceae family are well known abundant freshwater bacteria (Newton et al., 2011). The intermediate abundance group (cluster 4) was dominated by α-Proteobacteria and within the class, we found three bacteria that were relatively abundant and stable throughout the study period: *Phenylobacterium*, *Rhodobacter*, and *Candidatus* Megaira. This group also included *Limnohabitans* and *Flavobacterium*, which are common in many freshwater habitats, as well as *Aeromonas* and *Rheinheimera*, common aquatic bacteria which are known as potential fish pathogens (Janda and Abbott, 2010; Moore et al., 2014). Interestingly, several OTUs affiliated to the genus *Flavobacterium*, which is known to contain species that can degrade cyanobacterial hepatotoxins (Berg et al., 2009), were detected in this group.

Cluster 5 (OTUs found at intermediate abundance in the middle of the study period) was dominated by Proteobacteria, and the majority of OTUs within this phylum are β-Proteobacteria. However, this cluster was the most diverse group and included other phyla such as Bacteroidetes, Verrucomicrobia, Planctomycetes, Chloroflexi, and Actinobacteria. Interestingly, in this cluster we found an OTU identified as *Bdellovibrio*, one of the most well-known predatory bacteria (Pérez et al., 2016). The cluster also included another OTU of the genus *Cellvibrio* (OTU 45823), which appeared to be highly dynamic and peaked on early February, at the same time with the increase of the *Peridinium* biomass. Two other members of this group, *Flavobacterium* (OTU 40922) and *Silanimonas*, showed a different dynamic pattern where they peaked on early March and maintained a relatively high prevalence during March, at the same time with the biomass increase of *Microcystis* and *Peridiniopsis*.

### Fitting Between Environmental Parameters, Heterotrophic Bacteria and the Phytoplankton Community

**FIGURE 6:**
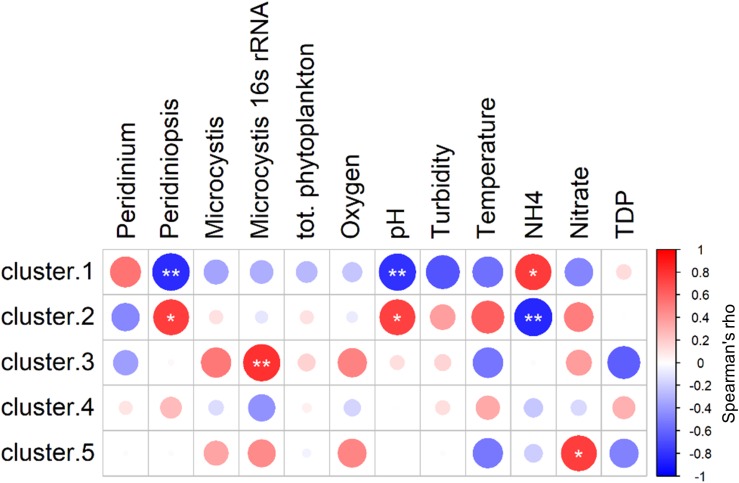
Spearman cross-correlations between centroids of each of the five clusters and environmental parameters. Cluster 1: descending abundance, Cluster 2: ascending abundance, Cluster 3: high abundance throughout the study period, Cluster 4: intermediate abundance throughout the study period, Cluster 5: OTUs found at intermediate abundance in the middle of the study period. *Peridinium* population (mm^2^/mm^3^), Peridiniopsis, *Peridiniopsis* population (mm^2^/mm^3^), Microcystis, *Microcystis* population (mm^2^/mm^3^), TDP, total dissolved phosphorus, (mg L^–1^). Starred Circles represent significant correlation (one star: 0.01 < *p* < 0.05, two stars: *p* < 0.01). Analysis was performed using ‘psych’ package in R (Revelle, 2013).

The results of the NMDS analysis (Figure 4) indicate that the heterotrophic community composition correlated with ammonia differently than with the other significant parameters, and that there are probably certain groups within the community that corresponded positively to ammonia but negatively to other parameters. To better characterize these relations we tested the correlations between the environmental parameters and the centroids of each of the five bacterial groups described above. Spearman cross-correlation analysis (Figure 6) revealed a close link between the dynamic bacterial groups (clusters 1 and 2) and the three factors: ammonia, pH and *Peridiniopsis* (Supplementary Table S5). We found that cluster 1 (descending abundance) corresponded positively to ammonia and negatively to pH and *Peridiniopsis*, whereas cluster 2 (ascending abundance) behaved inversely and showed positive correlation with pH and *Peridiniopsis* and negative correlation with ammonia. We also found that cluster 5 corresponded positively to nitrate, and interestingly, the high abundance group (cluster 3) correlated positively with *Microcystis* 16S rRNA gene copy number assessed by real time quantitative PCR, suggesting a potential link between abundant freshwater bacteria and *Microcsystis* cells.

**FIGURE 7:**
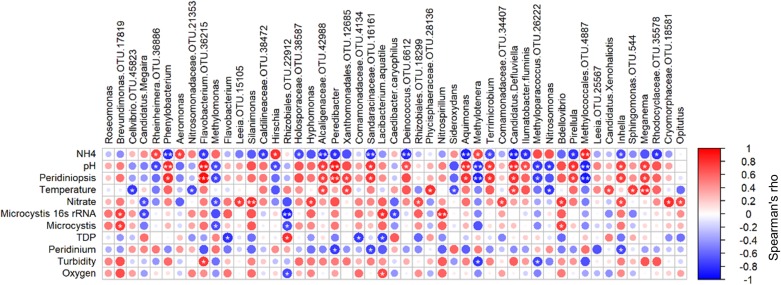
Spearman cross-correlations between relative abundance of significantly associated OTUs and environmental parameters. Starred circles represent significant correlation (one star: 0.01 < *p* < 0.05, two stars: *p* < 0.01). OTU numbers indicated for genera in which more than one OTU was identified. Peridiniopsis, *Peridiniopsis* population (mm^2^/mm^3^), Microcystis, *Microcystis* population (mm^2^/mm^3^), TDP, total dissolved phosphorus, (mg/l), Peridinium, *Peridinium* population (mm^2^/mm^3^). Analysis was performed using ‘psych’ package in R (Revelle, 2013).

In order to identify the key players within the heterotrophic microbial community, which may be linked to the environmental conditions, we tested Spearman cross-correlations between the environmental parameters and the 100 most abundant heterotrophic bacteria at the OTU level (Supplementary Table S6). Forty-nine of these OTUs were found to correlate significantly (*p* > 0.05) with one or more of the environmental parameters (Figure 7). Among them, 24 OTUs correlated significantly with pH and ammonia. Thirteen of these correlated positively with pH whereas only five had positive correlation with ammonia, including *Aeromonas* and *Rheinheimera*, as previously shown by Pinhassi and Berman (2003). Ammonia stands out from all the other environmental parameters, as it was found to negatively correlate with the highest number of OTUs (15 OTUs). *Peridiniopsis* and pH, which were found to co-vary (*r* = 0.93, *p* < 0.0001), correlated positively with 12 and 13 OTUs, respectively, with 11 overlapping OTUs. *Peridinium* was not positively associated with any of the tested OTUs, and was negatively correlated with three OTUs (*Peredibacter*, *Inhella* and the family Sandaracinaceae). Water temperature and nitrate were found to correlate positively with seven and nine OTUs, respectively. *Brevundimonas* (OTU 17819) was found to correlate positively with *Microcystis* (both microscopic counts and 16S rRNA gene copy number). *Microcystis* 16S rRNA gene copy number also correlated positively with *Nitrospirillum* and *Lacibacterium aquatile*, and interestingly, we found positive correlation between microscopic counts of *Microcystis*, and the predator taxa *Bdellovibrio*.

## Discussion

In this study we investigated the composition of the particle-associated bacterial community in a lake undergoing major physical, chemical and biological transitions. We focused on relatively large particles (>20 μm), collected by a plankton net from a large volume of water and integrating across the upper 15 m of the water column. The particles collected in this manner are highly heterogeneous and may include many of the dominant phytoplankton organisms in the lake (either as cells or as colonies), as well as some zooplankton and other organic and inorganic particles. Our aim was to identify correlations between the structure of the particle-associated bacterial community, the specific phytoplankton that contribute to the >20 μm particles, and various abiotic conditions. This approach, commonly used in studies of natural environments, suffers from several limitations. For example, measured correlations between microbes and a-biotic conditions do not necessarily imply that the conditions drive population structure, and correlations between organisms do not necessarily imply functional interactions (Paver et al., 2013). Additionally, assessments of bacterial community structure measured by 16S rRNA gene sequencing do not provide information about the absolute abundances of the organisms. Results may be affected by concentrations and quality of the DNA samples and by the abundance of other organisms. This may cause spurious correlations and confounding interpretation of microbial community dynamics (Schloss et al., 2011). For all these reasons, changes in the relative abundance of organisms, or observations of correlations between organisms or between them and a-biotic conditions, must be interpreted with caution. Nevertheless, our approach provides an important description of how the particle-associated microbial community changes over time during a dynamic period in the annual cycle of the lake, and provides testable hypotheses as to potential environmental and biotic factors affecting or driving the microbial population structure.

Our initial hypothesis was that the particle-associated community structure would be affected by the phytoplankton species and biomass. This hypothesis was based on previous evidence that phytoplankton host specific communities of associated heterotrophic bacteria (Goecke et al., 2013; Seymour et al., 2017). However, we found that the composition of the particle-associated bacterial community mainly corresponded with abiotic factors, such as pH, ammonia, water temperature and nitrate. This may suggest that the particulate fraction we sampled is comprised of a significant fraction of “non-selective” particles, which unlike phytoplankton do not host specific bacterial communities. Nevertheless, our results show that some specific bacterial populations correlated positively or negatively with certain phytoplankton species, and thus may indicate bacteria-phytoplankton association through direct or indirect relationships.

### Particle-Associated Bacteria Are Dominated by *Microcystis* and Two Members of α-Proteobacteria

Although *Microcystis* was not a major component of the phytoplankton biomass, it was the primary component of the particle-associated community assessed by 16S rRNA amplicon sequencing. The dominance of *Microcystis* throughout the sampling period (44% of total sequences) indicated a significant presence of *Microcystis* cells in the particles environment even when its biomass decreased.

Proteobacteria, as the relatively most abundant phylum within the heterotrophic community, are known to dominate particle-associated freshwater bacterial communities and cyanobacteria (Eiler and Bertilsson, 2004; Berg et al., 2009; Yang et al., 2017). α-Proteobacteria, the dominant class comprising the heterotrophic community, was previously regarded as abundant in marine pelagic habitats (Gilbert et al., 2012; Teeling et al., 2012), but rare in the pelagic zones of most lakes. Nevertheless, this group has previously been identified as associated with *Microcystis* colonies (Shi et al., 2009). The dominance of *Roseomonas* and *Brevundimonas* raises the question of whether these genera are ‘particle specialists,’ or just opportunists taking advantage of the mixing state of the lake. *Brevundimonas* is a known common freshwater genus (Newton et al., 2011) and has been previously found in association with “lake snow,” as well as with cyanobacteria and specifically with *Microcystis* (Schweitzer et al., 2001; Berg et al., 2009; Li et al., 2018). *Roseomonas* has been previously reported as an abundant component of freshwater populations (Garcia et al., 2013) and has been associated with cyanobacterial blooms (Bagatini et al., 2014) and with the cell surface of *Microcystis aeruginosa* (Eiler and Bertilsson, 2004). This suggests that these two bacteria may specialize in ‘particles’ and particularly in colonizing cyanobacteria in freshwater bodies.

Aquatic environments are highly dynamic and varied, hosting different microbial populations. Moreover, the identified population structure may differ based on the different tools and analysis methods used, as well as on the definitions of the particle-associated and free-living fractions. The dominance of cyanobacteria (specifically *Microcystis)* and Proteobacteria within the particle-associated community found here, was also recorded in a plateau lake in China and in several lakes in Germany (Allgaier and Grossart, 2006; Rösel et al., 2012; Yang et al., 2017). However, in most studies different phyla of bacteria were associated with the particles. Specifically, Bacteriodetes and Actinobacteria are often observed as major components of microbial communities (Allgaier and Grossart, 2006; Rösel et al., 2012; Morrison et al., 2017; Yang et al., 2017), whereas these organisms were relatively rare in our samples. This may be due to the different size cutoff for the particles used here (>20 μm) and in the studies mentioned above (3–5 μm). It is worth mentioning that the microbial population in marine environments continuously changes along a continuum of particle sizes (Mestre et al., 2017). To the best of our knowledge, this is the first study to identify such a high proportion of the two *α-*Proteobacterial genera described above, *Roseomonas* and *Brevundimonas*.

### Are Abiotic Conditions the Major Drivers of Population Structure in Particle-Associated Bacteria?

At the entire community level, we found that the particle-associated heterotrophic bacteria were mainly associated with abiotic factors, especially pH, ammonia and temperature, and only slightly with phytoplankton. Water temperature has been extensively documented as a major driver for the dynamics of freshwater particle-associated bacteria (Lindström et al., 2005; Rösel et al., 2012). In our results, the water temperature was significantly correlated to the microbial community composition, as water column temperature slightly, but consistently, increased throughout the sampling period.

The significant association between pH and particle-associated microbial populations has also been previously demonstrated in freshwater environments (Rösel and Grossart, 2012). This effect also extends to free-living populations (Allgaier and Grossart, 2006; Yang et al., 2017), as well as to the entire microbial community (Yannarell and Triplett, 2005). It is important to note that the strong correlation between pH and the particle-associated bacteria may be partially explained by the fact that phytoplankton, a major component of the particle fraction, causes an increase in pH through photosynthetic activity (Reid and Mosley, 2016). Indeed, multiannual monitoring of pH levels in the surface water of Lake Kinneret showed that during this period of the year pH typically increases, likely due to increased primary production (Nishri and Stiller, 2014). However, the positive association found here between pH and the total phytoplankton biomass was not statistically significant (*p* > 0.05), and thus it is likely that the effect of pH on particle-associated bacteria is direct.

Unlike most measured parameters, which showed positive correlations with bacterial OTUs, ammonia was more often negatively correlated with the individual bacterial OTUs. Moreover, ammonia was found to positively correlate with *Peridinium* (*r* = 0.55, *p* = 0.03), which constituted the most substantial component of the phytoplankton biomass during the early phase of the sampling period (February). This finding is consistent with early studies in Lake Kinneret which demonstrated that *Peridinium* preferentially utilizes ammonia over other N sources (Berman et al., 1984; Zohary et al., 2014). Interestingly, ammonia correlated positively with the group of descending abundance (cluster 1) which included several bacterial taxa involved with methane metabolism. The existence of putative methane-utilizing bacteria in the epilimnion layer is a rather surprising observation, as methane production in Lake Kinneret has only been recorded in sediment (Berman et al., 2014). Nevertheless, recent studies have convincingly shown that substantial methane production can occur in oxic freshwaters (Bogard et al., 2014; Donis et al., 2017), and this production can be attributed to cyanobacteria (Bižiæ-Ionescu et al., 2018b). This suggests that the presence of potential methane-utilizing bacteria in the particle-associated fraction may indeed be due to methane production within the particulate fraction. Yet, the presence of these bacteria may alternatively result from the movement of ammonia- and methane-containing water rising from the chemocline to the epilimnion, due to lake mixing and internal fluxes.

The only biotic factor found to significantly affect the composition of the microbial community was *Peridiniopsis*, which strongly co-variated with pH, and this might be partly due to *Peridiniopsis* preferring alkaline environments (Pollingher, 1987).

### Temporal Patterns of Certain Bacterial Populations Are Associated With Phytoplankton

Although we could not find a major link between phytoplankton and the particle-associated bacteria at the entire community level, we observed such associations between specific populations at the level of OTUs and clusters of OTUs sharing similar temporal dynamics. The nature of interactions between phytoplankton and associated bacteria during blooming events is complex and dynamic, and can range from mutualism, to commensalism, and even to parasitism. These interactions are ubiquitous and may affect the primary productivity in most ecosystems (Ramanan et al., 2016). Studies indicate that specific functional types of bacteria are associated with most algae, which exploit this unique habitat, in some cases also enhancing algal growth (Ramanan et al., 2015).

The dynamic structure of the heterotrophic bacterial community showed that there are some bacterial populations which are present and relatively stable throughout the study period (clusters 3 and 4), and dynamic bacteria which vary over time (clusters 1, 2, and 5). The high abundance bacterial group (cluster 3) correlated positively with *Microcystis* quantification, and at the OTU level we found that *Brevundimonas* (which, as discussed above, is a major component of cluster 3) co-varied with *Microcystis* as well. These findings support previous studies suggesting that *Brevundimonas* and *Roseomonas* are likely to be *Microcystis*-associated, as both of them were found to be common in *Microcystis* cultures (Bagatini et al., 2014; Li et al., 2018). The stability of the dominant bacterial group throughout the study period, and its covariation with *Microcystis*, are consistent with the findings of Woodhouse et al. (2018), which suggested that cyanobacterial associated microbial assemblages are comprised largely of stable dominant taxa.

At the OTU level, the correlation we found between *Bdellovibrio* and the biomass of *Microcystis* suggests that this predator may be associated with *Microcystis* colonies, probably due to the function of colonies as a surface niche, as surfaces are preferred by *Bdellovibrio* as habitat (Kelley et al., 1997). The gel-like matrix of *Microcystis* colonies may constitute a protective surface and provide the predators a rich source of prey bacteria. However, *Bdellovibrio* may also negatively affect *Microcystis*, as was demonstrated by Caiola and Pellegrini (1984), who showed cells of *Microcystis aeruginosa* that were infected and lysed by *Bdellovibrio*-like bacteria. Although we would expect a negative correlation in such a case, interaction based on the predation of *Microcystis* by *Bdellovibrio* may also display a predator-prey coupling, when rise in concentration increases the encounter rate between phytoplankton and their predators, thereby enhancing predator populations (Behrenfeld, 2014). The covariation between these two “key players” may also be due to the fact that the predation rate by *Bdellovibrio* may not be high enough to prevent *Microcystis* population growth. As this interesting relationship has not been sufficiently explored, and given the importance of understanding the factors driving *Microcystis* bloom dynamics, it is worth studying this interaction at more depth.

Examining the associations between *Peridiniopsis* and *Peridinium* with bacterial OTUs revealed that these two dinoflagellates potentially associate with different heterotrophic bacteria. *Peridiniopsis* was shown here to co-vary with many OTUs affiliated to a variety of phylogenetic groups. Many of these correlated positively with pH as well. Dissociating the effect of *Peridiniopsis* from that of pH is difficult, given that *Peridiniopsis* prefers alkaline environments (Pollingher, 1987), and may itself alter the pH due to its photosynthetic activity. Nevertheless, the observation that different bacteria are associated with *Peridiniopsis* compared to other phytoplankton species (e.g., *Microcystis* and *Peridinium*) suggests that the mechanism may be more specific than simply through the change in pH. Among the *Peridiniopsis*-associated OTUs were *Phenylobacterium*, *Flavobacterium*, *Pirellula*, and *Aquimonas*, which were previously shown to interact with phytoplankton (Riemann and Winding, 2001; Morris et al., 2006; Shi et al., 2009; Krohn-Molt et al., 2017). Surprisingly, *Peridinium* which strongly dominated the phytoplankton structure during the early phase of the study period, was not found to associate positively with any of the OTUs, but correlated negatively with three OTUs (*Peredibacter*, *Inhella* and an OTU affiliated to Sandaracinaceae). These findings raise the question whether these negative correlations are due to *Peridinium* feeding on the bacteria. Hitchman and Jones (2000) demonstrated high ingestion rates of bacteria by *Peridinium* during winter, when sunlight is low, and referred to this dinoflagellate as a mixotroph. Furthermore, since we only addressed here bacteria associated to particles >20 μm, these negative associations between *Peridinium* and specific bacterial taxa may be due to a mechanism in which *Peridinium* ingests other algae (Li et al., 1996), which in turn may already contain different bacterial species. Mixotrophy of *Peridinium* has been poorly studied in Lake Kinneret. Given the importance of *Peridinium* as a key primary producer in the lake (Zohary et al., 2012), mixotrophy by this organism and the manner which it might influence lake microbiota should be further addressed in future studies.

## Conclusion

Our study highlights the dynamic nature of a freshwater microbial community during the period of late winter and spring in Lake Kinneret. The results point to a strong link between abiotic factors and the structural patterns of the community at the entire particle-associated bacteria level. However, at the specific bacterial population level, we found associations with blooming phytoplankton species. The strong correlations presented here between the heterotrophic community and abiotic factors, rather than particle composition (phytoplankton), may be related to the nature of the sampled community, which was collected as integrated samples during a period of mild blooms of various phytoplankton (Figure 2). Analyzing a more pronounced phytoplankton bloom, such as those that previously occurred in Lake Kinneret (Sukenik et al., 2014), might have yielded a different picture than the one observed here, with a more significant link between the biotic conditions and the heterotrophic microbial community (Niu et al., 2011).

Correlated patterns may indicate a close link between phytoplankton and bacterial population dynamics, but they are not necessarily a result of direct causal relationships. While validation of causal relationships by structural equation modeling (SEM) is a useful tool to assess linkages between bacterial communities and phytoplankton in environmental observations studies (Liu et al., 2014), understanding the mechanisms that control direct interactions can only be achieved by experimental studies. Paver et al. (2013) combined time-series observations of relative abundances of specific phytoplankton and bacterial OTUs with phytoplankton manipulation experiments. Fourteen out of the 101 bacterial OTUs that exhibited positively correlated patterns of abundance with specific algal populations in time-series observations were enriched in mesocosms following incubation with phytoplankton. Their results support the hypothesis that, at least in some cases, phytoplankton succession directly affects changes in bacterial community composition. While the correlations discussed here cannot indicate causal relationships, they can be useful to infer patterns. Considering that particle-attached bacteria were analyzed here, we suggest that association of specific bacterial taxa with specific phytoplankton taxa may potentially control the dynamic patterns of the microbial community. Clearly, a combination of environmental observations and experimental manipulations are needed to gain a better understanding of the influence of phytoplankton on bacterial community dynamics.

## Data Availability

The datasets generated for this study can be found in the Sequence Read Archive (SRA) of the National Center for Biotechnology Information (NCBI) database under the BioProject ID: PRJNA543623.

## Author Contributions

OS-N, AS, and DS designed the experimental work and sampling regime. OS-N and AS performed the field sampling. OS-N and MO-L generated and analyzed the data. OS-N, AS, DS, and MO-L prepared the manuscript.

## Conflict of Interest Statement

The authors declare that the research was conducted in the absence of any commercial or financial relationships that could be construed as a potential conflict of interest.
