# Supplementary material for: Particle-Associated Microbial Community in a Subtropical Lake During Thermal Mixing and Phytoplankton Succession
Source: Front Microbiol. 2019 Sep 13;10:2142. doi: 10.3389/fmicb.2019.02142 (PMC6753980; doi:10.3389/fmicb.2019.02142)
Supplement: Supplementary file 4 [file Data_Sheet_1.docx]

Supplementary Material

# Supplementary Tables

**Supplementary Table S1.** Measured physical and chemical parameters of the upper water layer during hydrologic year of 2014-2015. Values are calculated means of integrated water column (0-15m). Average values (added in time points with missing data) marked in red. **(table 1.xlsx)**

**Supplementary Table S2.** Taxonomic identification and abundance of 16S OTUs defined at the level of 97% sequence similarity for the 15 sampling points. **(table 2.xlsx)**

**Supplementary Table S3.** Alpha diversity indices measurements pertaining to 16S OTUs for each time point.

| **Date** | **Shannon** | **Simpson** | **Species richness** |
| --- | --- | --- | --- |
| 11-Jan-15 | 4.49 | 0.96 | 807 |
| 18-Jan-15 | 4.00 | 0.92 | 764 |
| 25-Jan-15 | 4.34 | 0.96 | 632 |
| 1-Feb-15 | 3.35 | 0.91 | 427 |
| 8-Feb-15 | 3.57 | 0.89 | 462 |
| 15-Feb-15 | 4.82 | 0.98 | 868 |
| 22-Feb-15 | 3.59 | 0.89 | 621 |
| 1-Mar-15 | 3.70 | 0.90 | 609 |
| 8-Mar-15 | 3.84 | 0.92 | 591 |
| 15-Mar-15 | 3.79 | 0.92 | 557 |
| 22-Mar-15 | 4.03 | 0.94 | 603 |
| 29-Mar-15 | 4.06 | 0.91 | 744 |
| 15-Apr-15 | 4.36 | 0.95 | 607 |
| 19-Apr-15 | 4.36 | 0.95 | 620 |
| 26-Apr-15 | 4.86 | 0.97 | 880 |

**Supplementary Table S4**. Correlation coefficients and probability values for fitting environmental factors onto NMDS using the ‘envfit’ algorithm provided with the ‘vegan’ package (Oksanen et al., 2011).

| **Limnological variable** | ***r2*** | ***p*** |
| --- | --- | --- |
| NH4 | 0.6824 | 0.002 |
| pH | 0.6747 | 0.001 |
| Temperature | 0.5623 | 0.007 |
| Nitrate | 0.5345 | 0.017 |
| Peridiniopsis | 0.4251 | 0.032 |
| Oxygen | 0.4131 | 0.045 |
| *Microcystis*_16s rRNA | 0.3477 | 0.075 |
| *Microcystis* | 0.287 | 0.141 |
| *Peridinium* | 0.2191 | 0.238 |
| Total phytoplankton | 0.2477 | 0.197 |
| TDP | 0.1312 | 0.433 |

**Supplementary Table S5.** Pairwise Spearman’s correlation coefficients (r) between clusters centroids and environmental parameters generated using the ‘Psych’package (Revelle, 2013). Red highlighted values represent significant correlation (*p<0.05)*.

| **Lim. variable** | **cluster 1** | **cluster 2** | **cluster 3** | **cluster 4** | **cluster 5** |
| --- | --- | --- | --- | --- | --- |
| *Peridinium* | 0.546 | -0.464 | -0.371 | 0.107 | -0.011 |
| *Peridiniopsis* | **-0.825** | **0.750** | 0.021 | 0.264 | -0.014 |
| *Microcystis* | -0.357 | 0.114 | 0.525 | -0.139 | 0.354 |
| *Microcystis* 16s rRNA | -0.318 | -0.093 | **0.807** | -0.429 | 0.454 |
| Total phytoplankton | -0.271 | 0.111 | 0.175 | 0.054 | -0.043 |
| Oxygen | -0.229 | -0.075 | 0.489 | -0.168 | 0.479 |
| pH | **-0.803** | **0.748** | 0.125 | 0.005 | 0.000 |
| Turbidity | -0.664 | 0.379 | 0.161 | 0.125 | 0.014 |
| Temperature | -0.550 | 0.621 | -0.539 | 0.336 | -0.525 |
| NH4 | **0.768** | **-0.846** | -0.011 | -0.214 | -0.196 |
| Nitrate | -0.475 | 0.504 | 0.389 | -0.150 | **0.750** |
| TDP | 0.254 | -0.250 | 0.371 | -0.082 | 0.329 |

**Supplementary Table S6.** Pairwise Spearman’s correlation coefficients (r) between 100 most abundant OTUs and environmental parameters generated using the ‘Psych’ package. Red highlighted values represent significant correlation. **(Table 3.xlsx)**

# Supplementary Figures


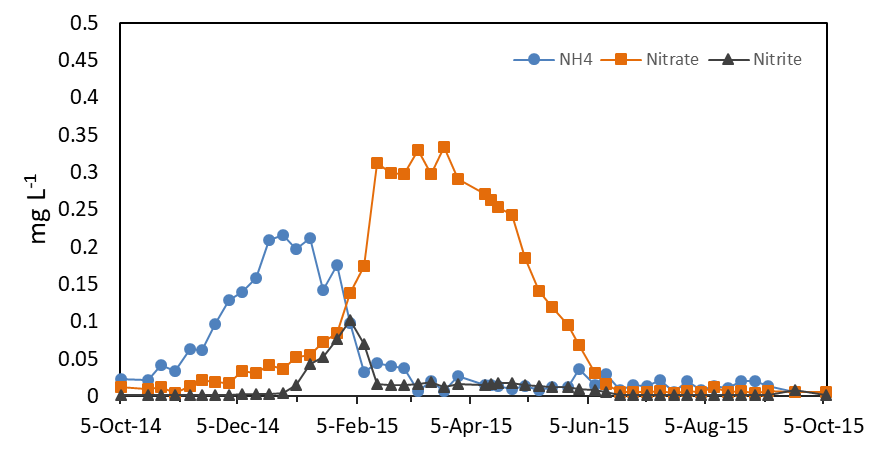


**Supplementary Figure S1.** Measurements of the three common forms of nitrogen in Lake Kinneret, over the whole hydrologic year of Oct-14 to Oct-15 (mgL^-1^) (Supplementary Table S1). Values represent depth average of 0-15 meters. Data obtained from Kinneret data center (IOLR), Dr. Werner Eckert, Dr. Yaron Beeri-Shlavin.


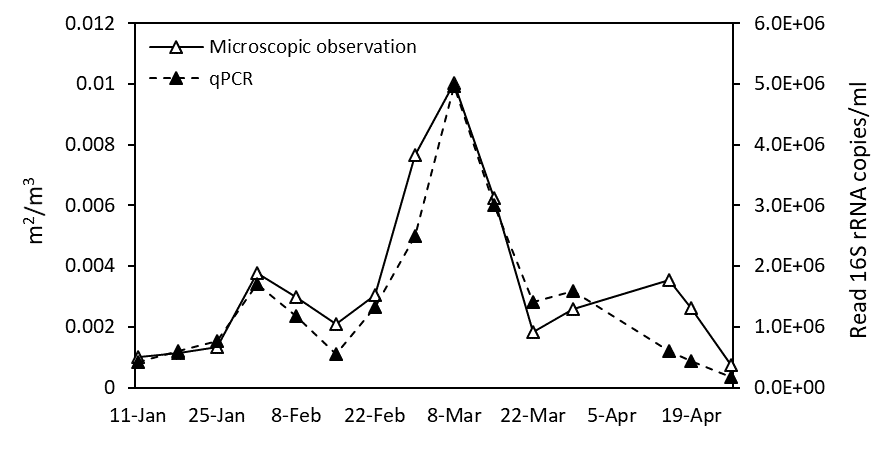


**Supplementary Figure S2.** *Microcystis* abundance by Real-Time quantitative PCR and by microscopic analysis.


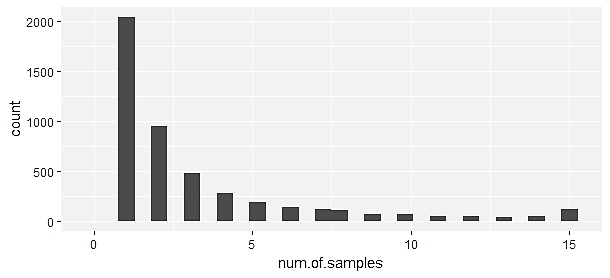


**Supplementary Figure S3.** Number of OTUs (y axis) as a function of the number of samples they are found in.
